# Learning Process of Gaze Following: Computational Modeling Based on Reinforcement Learning

**DOI:** 10.3389/fpsyg.2020.00213

**Published:** 2020-03-03

**Authors:** Mitsuhiko Ishikawa, Atsushi Senju, Shoji Itakura

**Affiliations:** ^1^Department of Psychology, Graduate School of Letters, Kyoto University, Kyoto, Japan; ^2^Japan Society for the Promotion of Science, Tokyo, Japan; ^3^Centre for Brain and Cognitive Development, Birkbeck, University of London, London, United Kingdom

**Keywords:** gaze following, reinforcement learning, computational modeling, infant internal state, communicative cues

## Abstract

Many studies have explored factors which influence gaze-following behavior of young infants. However, the results of empirical studies were inconsistent, and the mechanism underlying the contextual modulation of gaze following remains unclear. In order to provide valuable insight into the mechanisms underlying gaze following, we conducted computational modeling using Q-learning algorithm and simulated the learning process of infant gaze following to suggest a feasible model. In Experiment 1, we simulated how communicative cues and infant internal states affect the learning process of gaze following. The simulation indicated that the model in which communicative cues enhance infant internal states is the most feasible to explain the infant learning process. In Experiment 2, we simulated how individual differences in motivation for communication affect the learning process. The results showed that low motivation for communication can delay the learning process and decrease the frequency of gaze following. These simulations suggest that communicative cues may enhance infants’ internal states and promote the development of gaze following. Also, initial social motivation may affect the learning process of social behaviors in the long term.

## Introduction

Human infants show face preferences from the very early stages of life (Johnson et al., 1991; Valenza et al., 1996). Especially, newborns have sensitivity toward human eyes (Farroni et al., 2002). Studies have found that 2- to 5-day-old newborns discriminated between direct and averted gaze, and they were faster to make saccades to peripheral targets cued by gaze direction (Farroni et al., 2004). These studies suggest that infants may have a rudimentary form of gaze following from immediately after birth.

Many studies have suggested from which age infants start gaze following. The earliest precursor of gaze following was observed from 3 months (D’Entremont et al., 1997). In general, it is said that infants show gaze following from 6 months (Butterworth and Jarrett, 1991; Gredebäck et al., 2010).

Previous studies also investigated the contexts that trigger infant gaze following, and the results from these studies informed theoretical perspectives. For example, infants follow others’ gaze where accompanied by ostensive (communicative) cues (Senju and Csibra, 2008; Hernik and Broesch, 2019) or a highly attention-grabbing action (Szufnarowska et al., 2014). Ostensive cues or communicative cues were defined as signals showing communicative intent such as eye contact, while attention-grabbing cues did not include communicative intent but with visually salient movement. Other studies failed to find such a contextual modulation (Gredebäck et al., 2018). In the following section, we briefly describe the factors that have been argued to affect gaze following in infants.

### Communicative Cues

The theory of natural pedagogy suggests that infants follow others’ gaze because they refer to the topic of communication within the framework of ostensive-referential communication (Csibra and Gergely, 2009). This theory predicts that infant gaze following should be most prominent when it follows ostensive cues such as direct gaze and infant-directed speech (Csibra, 2010). Ostensive cues can signal that a partner interacting with an infant has communicative intent transferring knowledge (Csibra and Gergely, 2009).

In the first empirical study on the effect of ostensive signals on gaze following, Senju and Csibra (2008) showed that 6.5-month-old infants follow others’ gaze when it followed eye contact or infant-directed speech (communicative cues), but not when it followed attention-grabbing stimuli (e.g., non-social animation overlaid on top of the actor’s face). Based on this result, Csibra (2010) suggested that gaze following only occurs in the narrow context of ostensive cues early in life. Recently, Hernik and Broesch (2019) replicated this finding in 5- to 7-month-old infants developing in Vanuatu community, suggesting that the phenomenon is not fully dependent on a Western style of parenting. However, other studies showed conflicting evidence. For example, Szufnarowska et al. (2014) showed that 6-month-old infants followed others’ gaze direction when it followed a highly attention-grabbing, but not communicative, cue. In this study, total fixation duration to the model’s head during her action, attention-getting phase, was compared as an index of infant attention, and it was lower in the no-cues condition than in each of the other attention-grabbing conditions. Moreover, a recent study from the same group (Gredebäck et al., 2018) found that gaze following in 6-month-old infants was not different between ostensive (eye contact), attention grabbing (shivering), and no cue (no head movement), suggesting that infants follow others’ gaze without ostensive cues. The results suggest that infants’ gaze-following behavior is not fully dependent on the presence of preceding ostensive signal in some context.

### Infant Attention

Infant attention has been suggested as one of the factors affecting gaze following. As discussed above, Szufnarowska et al. (2014) showed that 6-month-old infants followed others’ gaze direction which followed highly attention-grabbing cues such as shaking a head horizontally. From these results, it was suggested that gaze following is based on infant attention.

However, such a viewpoint contradicts Senju and Csibra (2008) which did not observe gaze following in the situation with an attention-grabbing animation on the model’s face (see also Hernik and Broesch, 2019). In addition, as discussed above, Gredebäck et al. (2018) failed to show the effect of attention-grabbing cue on gaze following. The results suggest that infant gaze-following behavior cannot be induced only by attention-grabbing stimuli.

Many studies have investigated infant gaze following; however, the results were not consistent. For example, looking times to an actor’s face were different in each study. Previous studies used looking time to the actor’s face to measure infant attention. Szufnarowska et al. (2014) showed that eye contact attracted more infant attention than a no-cues condition. On the other hand, Gredebäck et al. (2018) indicated that infants looked at the actor in the shivering condition (social and non-ostensive cue) longer than in both the eye-contact condition and the no-cues condition. Although some studies suggested that infant attention affects gaze following, looking times to an actor’s face do not always correlate with gaze following (Gredebäck et al., 2018; Ishikawa and Itakura, 2019), suggesting that looking time may not capture infant engagement in the task.

### Correlation Between Communicative Cues and Infant Internal State

Ishikawa and Itakura (2019), by contrast, used heart rate as an alternative measurement of infants’ internal states and suggested that (a) infants’ gaze-following behavior is related to infants’ physiological arousal and (b) looking time to the actor’s face may not predict gaze following or infant internal state including attentional and physiological arousal measured by heart rate. In this study, there were three conditions, eye-contact, no-cues, and shivering conditions. The results of their study revealed that eye contact enhanced heart rate levels in 10-month-old infants, although there was no difference of looking time to the actor’s face across conditions. Also, infants showed gaze following above chance level only with eye contact, consistent with a claim derived from the theory of natural pedagogy. Interestingly, infant heart rate levels during an actor’s action predicted later gaze following in situations both with and without communicative cues and partially mediated between the conditions of communicative cues and gaze following. It has been suggested that physiological arousal is related to sensitivity and responsiveness to external stimuli (Aston-Jones et al., 1991, 1999). Infant studies have also shown results supporting the relation between physiological arousal and attentional state (Wass et al., 2016; de Barbaro et al., 2017). Also, empirical studies have shown that affective states and reward expectations can be reflected in physiological arousal (Critchley et al., 2005; Tummeltshammer et al., 2019). Because it is difficult to define which factors induce infant physiological arousal in gaze-following situations, here we use a broad concept of internal state, which could be measured by neurophysiological measurements.

Therefore, it can be considered that communicative cues affect the infant’s internal state, which may be reflected in physiological arousal, and both communicative cues and the infant internal state may promote infant gaze following.

### Importance of Computational Models

Theories of the emergence of gaze following have been examined in behavioral experiments. However, the results of empirical studies were inconsistent, and the mechanism of gaze following remains unclear. This could partly be because it is difficult to include all factors related to gaze following and conduct many trials in infant behavioral experiments. Also, individual differences were difficult to assess with the small numbers of participants typically included in empirical infant studies. We conducted computational modeling of infant gaze following in an attempt to address these issues and complement empirical studies.

Computational modeling allows us to examine what is difficult to conduct with real infants in experimental settings, and it is very useful to theorize human development (Triesch et al., 2006). Triesch et al. (2007) used computational modeling to simulate the emergence of gaze following. Because reward-driven learning can be found from a very early developmental stage (Floccia et al., 1997) and suggested as a principal learning mechanism (Sutton, 1988), they applied reinforcement learning to modeling and suggested how gaze following emerges in the mother–infant interaction. However, their model did not include communicative cues, and their simulations were mainly based on the theory of mirror neuron system (Triesch et al., 2007).

It has been shown that communicative cues facilitate infant learning in the social context (Csibra and Gergely, 2009). Also, it has been suggested that looking at the same object with another person is rewarding for infants (Moore and Corkum, 1994; Mundy, 1995). Thus, in the learning process of gaze following, contextual information such as communicative cues may affect reinforcement. The modeling is not informative as to the conflicting results reported in recent empirical studies; more precisely, communicative cues and infant internal states were not taken into account in previous simulation studies (Triesch et al., 2006, 2007). Computational modeling with the factors examined in experimental settings may offer a new perspective on the mechanism of gaze following.

### The Purpose of This Study

In this study, we simulated the learning process of infant gaze following and suggest a feasible model according to the results of empirical studies. It has been shown that reinforcement learning is the fundamental learning process in humans and is neurally plausible (Dayan et al., 2001; Holroyd and Coles, 2002); therefore, we applied reinforcement learning to simulate the early learning process of gaze following in infants and examined how infant internal states and communicative cues affect gaze following. Although computational modeling cannot compare models’ feasibilities statistically, it is suggested that computational modeling may be particularly helpful to theorize because it can easily monitor all changes in the model (Triesch et al., 2006). To theorize about the development of gaze following, we compared three models, the communicative cue model, the communicative cue and infant internal state model, and the model in which communicative cues enhance infant internal state.

## Methods

### Environment and Parameters

In the previous computational modeling of gaze following (Triesch et al., 2006, 2007), learning environment was posited that an infant and a caregiver interact with a number of objects, not only with a gaze target and a distractor. In a more complex way, they posit that caregiver’s gaze direction was not always perfectly aligned with the caregiver’s head orientation. Also, in the previous model, object locations were randomly distributed. Because they focused on creating a general model of gaze following interactions, and not on examining the effects of communicative cues and infant internal states, their simulation included the process of infant visual system affected by object saliency, caregiver’s saliency, or infant visual field.

**FIGURE 1:**
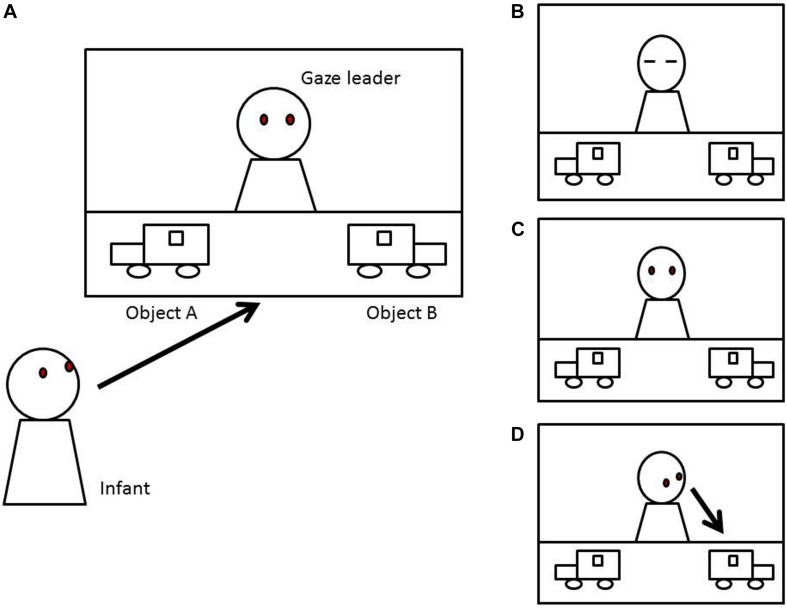
Learning environment: an infant watches the situation with an actor and two objects **(A)**. The actor closes his eyes in the initial phase **(B)**. Next, the actor shows different actions such as opening his eyes **(C)** and then looks toward one of two objects **(D)**.

In order to examine the effects of communicative cues and infant internal states, we simplified experimental situations to be based on previous empirical studies (Senju and Csibra, 2008; Szufnarowska et al., 2014; Gredebäck et al., 2018; Hernik and Broesch, 2019; Ishikawa and Itakura, 2019; Figure 1), and actor’s gaze direction was always consistent with the head orientation. An actor looked toward one of two objects with or without communicative cues. Two objects had the same saliency, and there was no looking bias. Infants were postulated to look to one of two objects 100% at the end of a trial. There were two considerable options of behavior for the infants, (a) following the actor’s gaze or (b) looking toward one of the two objects randomly. The learning process was simulated by the *Q*-learning algorithm, which is one of the most popular reinforcement learning algorithms (Watkins and Dayan, 1992). The *Q*-learning algorithm is as follows in (1):

(1) $$Q_{\left[t+1\right]}=Q_{\left[t\right]}+\alpha \times \left(R\times P_{\left[r\right]}-Q_{\left[t\right]}\right)$$

In *Q*-learning, a learned decision policy is determined by the behavioral value function, described as *Q*. To limit infant learning in a trial, learning rate “α” was the same in all simulations. Also, the reward probabilities (*P*r) were 100% for gaze following and 50% for random looking. This is because we posit that infants feel rewarded when they look at the same object as a model (Moore and Corkum, 1994; Triesch et al., 2006). The reward value (*R*) was 1 in all the simulations. Each simulation was continued up to 2,000 trials. We compared how the behavioral values of gaze following were updated during 2,000 trials. We adopted a “soft-max” strategy for selecting the infants’ actions in all simulations. In the soft-max strategy, the worthiest action is still given the highest selection probability, but all the others are ranked and weighted according to their value estimates (Sutton and Barto, 1998). All the parameters used in the simulations are shown in Table 1.

**TABLE 1:** Overview of model parameters and their allowed ranges.

**Symbol**	**Explanation**	**Range**
*t*	Number of trials	[1, 2000]
*Q*(A)	Behavioral value of random looking	(0, 1)
*Q*(B)	Behavioral value of gaze following	(0, 1)
*P*(A)	Probability of random looking	(0, 1)
*R*	Reward value	1
*P*(r)	Probability of reward	0.5 or 1
alpha	Learning rate	0.005
*S*	Infant state	[0, 1]
*D*s	Infant default state	[0, 1]
*C*	Other’s communicative intent	[0.5, 1.5]
*M*	Motivation for communication	constant

### Communicative Cue Model

With communicative cues, it is considered that infants can expect that an interacting partner is transferring knowledge (Csibra and Gergely, 2011). Therefore, we set up that communicative cues modulated the subjective reward probability. We added a variable named “*C*” (communicative cue) into the QL formula. The formula with communicative cues is shown in (2):

(2) $$Q_{\left[t+1\right]}=Q_{\left[t\right]}+\alpha \times \left(R\times P_{\left[r\right]}\times {\text{C-Q}}_{\left[t\right]}\right)$$

*C* takes a random number between 0.5 (low communicative intent) and 1.5 (high communicative intent) in each trial. Because *C* is an external factor and it is conceptually highly depending on context, *C* was taken from a flat uniform distribution (mean = 1).

### Communicative Cue and Infant Internal State Model

Infant attention has been argued to affect the perception and learning of the external environment (Rose et al., 1999; Tellinghuisen et al., 1999; Oakes et al., 2000). We set up that infant internal states modulate learning rate in QL. We added a variable named “*S*” (state) into the QL formula. The formula including infant internal states is shown in (3):

(3) $$Q_{\left[t+1\right]}=Q_{\left[t\right]}+\alpha \times S\times \left(R\times P_{\left[r\right]}\times {\text{C-Q}}_{\left[t\right]}\right)$$

*S* takes a random number between 0 (inattentive) and 1 (highly attending) in each trial. *S* reflects an internal state, and it should be stable around resting state most of the time; therefore, *S* was taken from a normal distribution (mean = 0.5, σ = 0.16). *S* modulates the learning rate so it cannot exceed 1 because α is the limit of infant leaning in a trial.

### Communicative Cues Enhancing the Infant Internal State Model

Formula (3) postulates that communicative cues and the infant internal state are independent of each other. In addition, we simulated a model that communicative cues enhance infant internal states, following the finding of Ishikawa and Itakura (2019).

**FIGURE 2:**
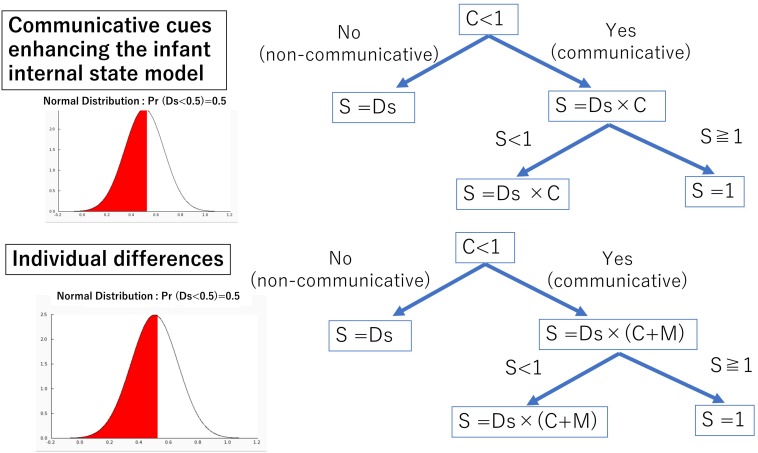
Decision tree of variable model parameters.

To simulate that, we set up the “Default state” (Ds), which is the infant internal state before the effect of communicative cues. Ds takes a random number between 0 (inattentive) and 1 (highly attending) from a normal distribution (mean = 0.5, σ = 0.16) in each trial. Here, *S* takes a number which is Ds modulated by *C* (Figure 2). If *C* is less than 1, *S* will be Ds × 1. Otherwise, if *C* is greater than 1, *S* will be Ds × *C*. Ishikawa and Itakura (2019) indicated that communicative cues enhance infant physiological arousal, but “no cues” do not affect the infant internal state. Therefore, we set up that if *C* is less than median (1), the default state is not modulated. In addition, we posited that if Ds × *C* is more than 1, *S* will be 1. As mentioned above, *S* modulates the learning rate so it cannot exceed 1 because α is the limit of infant learning in a trial; so, if Ds × *C* is more than 1, *S* takes 1.

## Experiment 1 Results and Discussion

**FIGURE 3:**
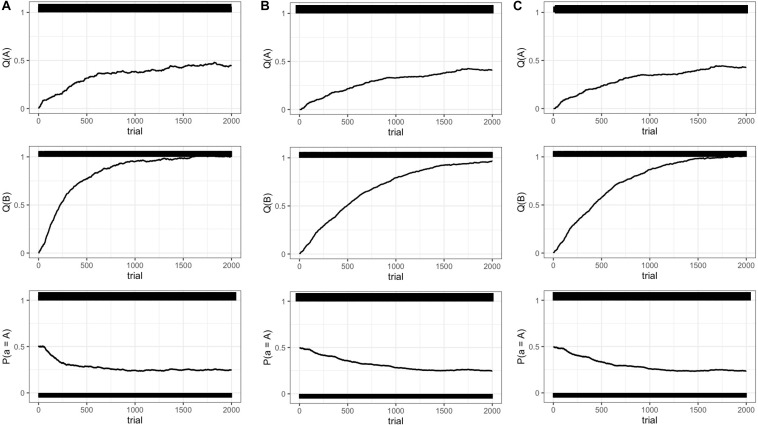
The results of Experiment 1 in 2,000 steps. **(A)** Communicative cue model. **(B)** Communicative cue and infant internal state model. **(C)** Communicative cue enhancing infant internal state model. *Q*(A) the behavioral value of random looking; *Q*(B) the behavioral value of gaze following; *P*(A) the probability of random looking predicted by a soft-max strategy.

Each experiment starts with all weights set to zero, and the models are simulated for a total of 2,000 time steps. The results are shown in Figure 3. All of these models were set up not to affect the optimal value of gaze following, thus all behavioral values after convergence were the same. In the model which only contains communicative cues, infant learning progressed at the same rate because infant internal state was kept constant through the simulation (Figure 3A, middle panel). Therefore, with high communicative intent, behavioral value drastically increased and the learning process was the most efficient. In the *Q*-learning, the speed of convergence means time taken to find a near-optimal behavioral choice. The behavioral value of gaze following was converged around 1,600 trials (Figure 3A, middle panel). However, infant internal states were kept at the same level in this simulation, which would fail to simulate more realistic infant behavior affected by internal states.

With the addition of the infant internal state which is independent from communicative cues, the convergence of behavioral value took over 2,000 trials (Figure 3B, middle panel). Here, infant internal states affected the learning rate. Therefore, although communicative cues were presented, if the infants were inattentive, they did not learn the behavioral value so much. In this model, infant learning is highly dependent on infant internal states.

In the model in which communicative cues enhanced infant internal states, the learning process was more efficient, converging around 1,750 trials (Figure 3C, middle panel). From the perspective of learning efficiency, the model with only communicative cues was the most efficient, but given that infant internal states are highly unlikely to be constant, the third model may be the most feasible to explain infant gaze following. Support for this model could also come from a recent empirical study that communicative cues can enhance the infant physiological state (Ishikawa and Itakura, 2019).

In addition, the Supplementary Material shows the learning process within 100 trials to observe how these models affect the short-term learning process. Communicative cues can directly modulate the subjective expected value, and they drastically update behavioral value in a very short term. On the other hand, if infant internal states change trial by trial, the behavioral value is updated gradually. Also, in the model with communicative cues enhancing states, the learning rate is increased in a trial with a high expected value, and, as a consequence, behavioral value was observed to be enhanced soon.

This simulation shows a possible learning process of gaze following. As a result, the model with communicative cues enhancing infant internal state can be considered the most feasible to describe infant learning. In this study, we indicated that communicative cues may affect learning drastically in the short term; however, they would not affect the emergence of gaze following so much after the behavioral value was converged. Notably, after the behavioral value of gaze following was converged, it was predicted that the infants followed the other’s gaze about 75%, regardless of communicative cues (Figure 3, lower panels). It is consistent with Gredebäck et al. (2018) who showed that infants show gaze following in situations both with and without communicative cues. These results of the simulation demonstrate that a prior history of social learning, either within the experimental context or in real-life experience, may be an important factor in gaze following in empirical experiments.

In Experiment 1, we posited the learning process of gaze following in the experimental setting used in many empirical studies but did not include one important factor: individual difference. It has been reported that individual differences of gaze following can be observed in experimental situations (Morales et al., 1998, 2000; Brooks and Meltzoff, 2005). In Experiment 2, we applied the model with communicative cues enhancing states to simulate individual differences of gaze following.

## Experiment 2: Modeling Individual Differences

There is some evidence indicating individual differences of gaze following (see the review by Frischen et al., 2007). For example, sex differences in the sensitivity to other people’s eye gaze can be detected from an early developmental stage. Lutchmaya et al. (2002) showed that in 12-month-old infants, male infants made less eye contact than female infants. Also, male infants looked toward faces less than females (Connellan et al., 2000; Lutchmaya and Baron-Cohen, 2002).

Also, autistic-like traits correlated with gaze cueing effects (Bayliss and Tipper, 2005; Bayliss et al., 2005). Children with autism spectrum disorder (ASD) are inattentive to social stimuli, and this means that they may lack adequate social learning experiences (Mundy and Neal, 2000; Schultz, 2005). One possible explanation for this phenomenon is the atypical development in social motivation in ASD (Chevallier et al., 2012). Some also argue that atypical development in social motivation in individuals with ASD is related to the atypical development of social reward processing (Bartz et al., 2011; Modi and Young, 2012; Dubey et al., 2015), although others did not find differences in the reinforcement value of social stimuli between individuals with and without ASD (e.g., Ewing et al., 2013; Vernetti et al., 2018). Overall, there is a theoretical and clinical interest in the possible influence of social motivation on the development of social attention and learning.

Here, we posit individual differences of motivation for communication. For example, autistic people and patients with social anxiety actively avoid to make eye contacts (Corden et al., 2008; Wieser et al., 2009). Thus, motivation for communication could be negative. We added a variable named “*M*” (motivation) into the model with communicative cues enhancing states. *M* can be considered to affect subjective evaluation of communicative cues; therefore, it modulates *C* directly (Figure 2). If *C* + *M* is less than 1, *S* will be Ds × 1. Otherwise, if *C* + *M* is more than 1, *S* will be Ds × (*C* + *M*). In addition, if Ds × (*C* + *M*) is more than 1, *S* will be 1. The formula including infant internal states is described as follows in (4):

(4) $$Q_{\left[t+1\right]}=Q_{\left[t\right]}+\alpha \times S\times \left(R\times P_{\left[r\right]}\times \left(C+M\right)\text{-}Q_{\left[t\right]}\right)$$

*M* is a fixed number because motivation for communication can be considered as a trait for each individual. If *M* takes a negative value, it means that infants actively avoid communication. On the other hand, if *M* takes a positive value, infants engage communication more than average. Therefore, when infants have standard motivation for communication, *M* takes 0 in this model. Here, we examined how the individual differences in *M* affect the updating of gaze-following value.

## Experiment 2 Results and Discussion

**FIGURE 4:**
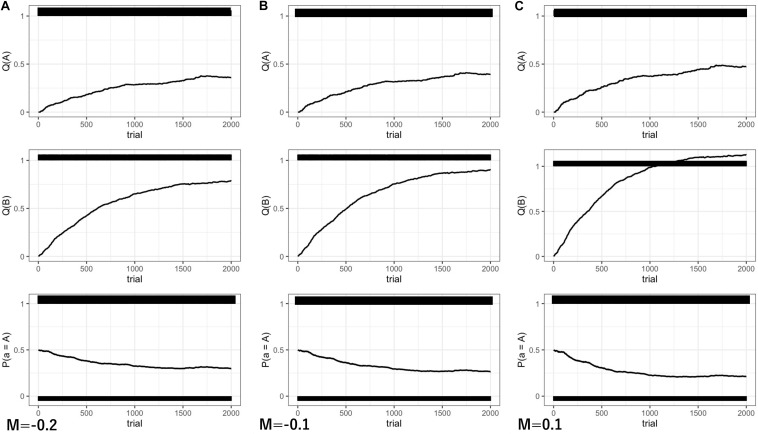
The results of Experiment 2 in 2,000 steps. **(A)** *M* = –0.2; **(B)** *M* = –0.1; **(C)** *M* = 0.1. *Q*(A) the behavioral value of random looking. *Q*(B) the behavioral value of gaze following. *P*(A) the probability of random looking predicted by a soft-max strategy.

The model was simulated for a total of 2,000 time steps. The results are shown in Figure 4.

Because behavioral values are converged to expected optimal values in *Q*-learning, the convergent value of gaze following was modulated by the degree of *M*. With *M* less than 0 (low motivation for communication), the subjective value of gaze following was undervalued and the expected reward value was decreased (Figures 4A,B, middle panels). For example, when M takes −0.2, the expected reward value of gaze-following behavior becomes 0.8, and the convergent value is also decreased to 0.8 (Figure 4A, middle panels). As a result, the probability of gaze following was also decreased slightly (*M* = −0.2: *P*(B) = 70%, Figure 4A, lower panel).

Here, we posit that motivation for communication may be behind individual differences in gaze following. Twomey and Westermann’s (2018) infant computational modeling study suggested that infants are intrinsically motivated to select information that maximizes learning. In the context of learning in gaze following, it can be considered that interacting with others would maximize information to learn about the environment. For example, because gaze direction can help infants to associate words and objects, the development of gaze following affects later language development (Morales et al., 1998; Brooks and Meltzoff, 2005). Therefore, low motivation for communication can delay the social learning process in infants.

## General Discussion

In our simulation, the model, in which communicative cues affect infant internal states, emerged as the most feasible to explain infant gaze following. It shows more efficient learning than the model in which infant internal states are independent of communicative cues, and it shows comparable efficiency with the less realistic model which assumes that infant internal states are constant. The model is also consistent with the results of empirical research which examined the relation between communicative cues and infant physiological states (Ishikawa and Itakura, 2019).

The model hypothesizes that physiological arousal, at least partially, mediates the influence of ostensive signals on infant gaze following. The theory of natural pedagogy claims that human communication makes it possible to efficiently convey knowledge; in other words, communication can promote social learning (Csibra and Gergely, 2011). As communicative cues such as eye contact elevate physiological arousal (Nicholls and Champness, 1971; Helminen et al., 2011; Hietanen, 2018), and high physiological arousal is hypothesized to promote the learning process and enhances memory (Kleinsmith and Kaplan, 1963; Eysenck, 1976) particularly memory consolidation (LaBar and Phelps, 1998), infant learning may be more efficient with communicative cues partly because it enhances infant internal states, which we observe as physiological arousal.

Our model also showed that low motivation for communication can delay the learning process of gaze following. The model thus suggests that individuals with low social motivation, possibly including those with ASD, may be delayed in the learning process of gaze following. It is consistent with an empirical study reporting that neural sensitivity to dynamic eye gaze in infants aged 6–10 months old is associated with later emerging autism (Elsabbagh et al., 2012), even though they show typical gaze-following behavior (Bedford et al., 2012). Further, gaze following in infants predicts later language development and theory of mind skills (Brooks and Meltzoff, 2005, 2015). Initial social motivation may affect the learning process of gaze following, and as a result, the development of other social cognitions might also be affected.

Through the experiments, we simulated how infants’ learning process of gaze following is affected by communicative cues, infant internal states, and social motivation. Our model was designed to offer simple simulation with reinforcement learning. Thus, the decision process of gaze following was only dependent on learned behavioral value. Following the results for the prediction of gaze following, after the behavioral value was converged, it can be predicted that gaze following emerges 75% in any situation, with or without communicative cues. It is consistent with Gredebäck et al. (2018), who indicated that infants show gaze following more than chance level (50%) in all experimental situations with or without communicative cues, and they suggested that infant gaze following is not dependent on communicative cues. Our simulation, consistent with the results of Gredebäck et al. (2018), indicates that after infants have experienced a sufficient number of gaze-following situations, infants tend to follow the other’s gaze direction with or without preceding ostensive signals. Effects of communicative cues on social interactions have been mainly studies in infants. However, some studies have shown that toddlers can understand other’s communicative intentions without ostensive signals such as eye contact and infant-directed-speech (IDS) (e.g., Moore et al., 2013). Social experiences in development may affect the engagement of interactions without communicative cues.

Note that the model has not accounted for other contextual information which affects gaze following, such as the other’s social status, familiarity, facial expressions, or object pleasantness (Deaner et al., 2006; Dalmaso et al., 2011; Kuhn and Tipples, 2011). It is crucial for future studies to include more generalized contextual modulation because a recent study suggested that infants could use contextual information to guide their visual attention (Tummeltshammer and Amso, 2018). Further theoretical work is needed, which can account for how human infants, as well as older children and adults, decide to follow the other’s gaze direction based on many kinds of contextual information to fully describe, explain, and predict gaze following in more naturalistic settings. Another limitation of this study is a lack of empirical data of developmental trajectory. This study theoretically simulated how external and internal factors affect the learning process of gaze following and chose a feasible model according to results of empirical studies in experimental settings. Computational modeling is useful to theoretically simulate and observe how behavioral models work; however, it cannot decide which models capture the development in real-world situations. To understand the development of gaze following, it is necessary to compare with longitudinal data measuring gaze-following behavior.

To conclude, the results of the simulation presented in this paper suggest that the model in which communicative cues affect infant internal states is feasible to describe the learning process of gaze following. Also, with this reinforcement learning model, we succeeded in simulating how social motivation affects the development of gaze following and showed that low motivation for communication delayed the learning process of gaze following. In future works, other factors affecting the learning mechanism should also be included (e.g., tolerance to reversal learning). Computational modeling of the experimental setting can be helpful to give new insights into gaze following in infants.

## Data Availability Statement

The datasets generated for this study are available on request to the corresponding author.

## Author Contributions

MI designed the study, conducted computer simulation, and wrote the initial draft of the manuscript. All authors have contributed to interpretation, critically reviewed the manuscript, and approved the final version of the manuscript.

## Conflict of Interest

The authors declare that the research was conducted in the absence of any commercial or financial relationships that could be construed as a potential conflict of interest.
