# Supplementary material for: Learning Process of Gaze Following: Computational Modeling Based on Reinforcement Learning
Source: Front Psychol. 2020 Mar 3;11:213. doi: 10.3389/fpsyg.2020.00213 (PMC7063100; doi:10.3389/fpsyg.2020.00213)
Supplement: Supplementary file 1 [file Image_1.PDF]

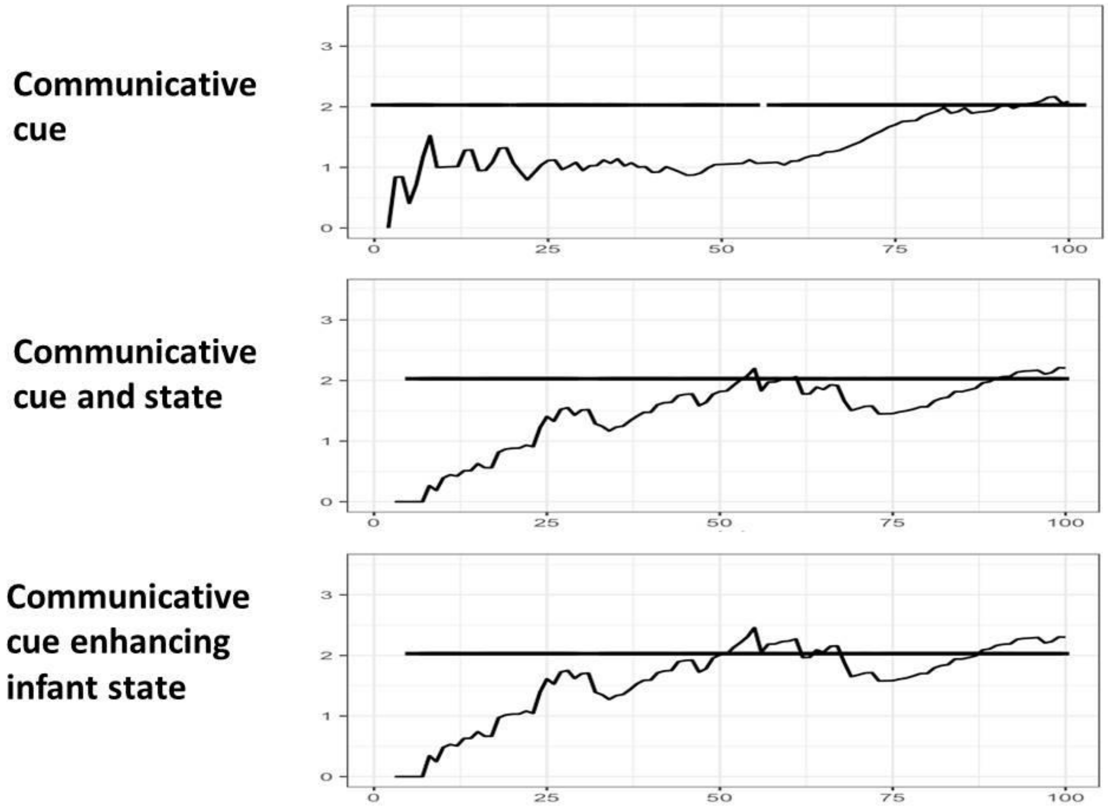

Supplementary: Fluctuation of gaze following value up to 100 trials [Q(B)/Q(A)].
